# Asymptomatic Children as a Missing Link in Preventing COVID-19 Transmission

**DOI:** 10.34172/jrhs.2024.149

**Published:** 2024-06-01

**Authors:** Iraj Sedighi, Roya Raeisi, Jalaleddin Amiri, Zohreh Shalchi, Manoochehr Karami, Farid Azizi Jalilian, Ali Teimoori, Nastaran Ansari, Jalaledin Bathaei, Mohammad Hashemi

**Affiliations:** ^1^Department of Pediatrics, School of Medicine, Hamadan University of Medical Sciences, Hamadan, Iran; ^2^Department of Epidemiology, School of Public Health and Safety, Shahid Beheshti University of Medical Sciences,Tehran, Iran; ^3^Department of Virology, School of Medicine, Hamadan University of Medical Sciences, Hamadan, Iran; ^4^Deputy of Health, Hamadan University of Medical Sciences, Hamadan, Iran

**Keywords:** Asymptomatic, Child, Transmission, COVID-19

## Abstract

**Background:** Investigating the prevalence of the coronavirus disease 2019 (COVID-19) infection in asymptomatic children who have been in close contact with symptomatic individuals is instrumental for refining public health approaches, protecting vulnerable populations, and mitigating the broader impact of the pandemic. Accordingly, this study aimed to evaluate the incidence of COVID-19 infection in asymptomatic children who had been in close contact with parents exhibiting COVID-19 symptoms.

**Study Design:** A cross-sectional study.

**Methods:** The present cross-sectional study was conducted on 175 asymptomatic children who had been in close contact with COVID-19 confirmed cases in Hamadan County from March 2021 to August 2021. Reverse transcription polymerase chain reaction (RT-PCR) testing was performed on all asymptomatic children who had been in close contact with an individual with COVID-19. Furthermore, multiple logistic regressions were conducted to determine the predictors of COVID-19 transmission from family members to children.

**Results:** Out of the 175 children in close contact with index cases, 53 (30.29%) tested positive for COVID-19 through PCR. Regarding factors related to the index case, male cases (Adjusted odds ratio [AOR]=2.29; 95% confidence interval [CI]: 1.03-5.09, *P*=0.041), rural dwellers (AOR=3.22; 95% CI: 1.02-10.16, *P*=0.046), illiterate cases (AOR=8.45; 95% CI: 1.76-40.65, *P*=0.008), and cases presenting with nasal congestion symptoms (AOR=9.12; 95% CI: 2.22-37.40, *P*=0.002) were more prone to transmitting the virus to children who had close contact with them.

**Conclusion:** The findings of the present study suggested that asymptomatic COVID-19 infection in household contacts is significant in children who were in close contact with a COVID-19-positive patient. Therefore, it is crucial to continue to monitor this group closely.

## Background

 In response to the challenges posed by the coronavirus disease 2019 (COVID-19 pandemic), various studies are underway to determine the most effective approaches for reducing the spread of COVID-19. It seems that healthcare systems worldwide should proactively work to minimize the transmission rates of COVID-19 infection following close contact.^1-3^

 According to guidelines from the World Health Organization (WHO), adherence to established protocols is strongly advised for individuals worldwide. Additionally, national healthcare systems should prioritize the identification of individuals with COVID-19 through close contact tracing.^4^ Although the most common finding among children was a cough, it was presented in 73% of cases, and the number of asymptomatic cases was considerable.^4^ However, new evidence highlights the pre-symptomatic transmission of COVID-19. In addition, there is growing evidence emphasizing the role of asymptomatic infections in the transmission of the disease.^5^

 Current research findings suggest that a substantial portion, ranging from 15% to 45%, of COVID-19 cases are asymptomatic, which are classified into two categories: those who remain entirely asymptomatic (referred to as asymptomatic) and those who are initially asymptomatic but later develop symptoms (referred to as pre-symptomatic).^6^ Recent studies have demonstrated that in both pre-symptomatic and asymptomatic cases, reverse transcription polymerase chain reaction (RT-PCR) is positive with similar viral loads.^7^ Furthermore, a significant proportion of asymptomatic COVID-19 cases are children, who can potentially serve as carriers and spreaders of the virus.^8^ Notably, no study has yet investigated the effect of commonly used drugs on the treatment of COVID-19 ^9,10^ Additionally, recent studies have indicated that circulating new variants of the virus may potentially impact the efficacy and effectiveness of vaccines. Consequently, there remains a focus on conventional methods to prevent COVID-19 transmission.^11,12^

 Due to cultural conditions in Iran, children are kept in large families and under quarantine conditions, resulting in continued contact with other extended family members outside the home, especially their elderly grandparents. New reports confirm the transmission of the virus from asymptomatic children to other family members and even individuals who have had contact with others outside the home.^13,14^

 Since children are not yet fully vaccinated against COVID-19, when compared with adults, it is important to investigate the prevalence of COVID-19 infection among asymptomatic children. Investigating the prevalence of COVID-19 infection in asymptomatic children who have had close contact with symptomatic individuals is instrumental for refining public health approaches, protecting vulnerable populations, and mitigating the broader impact of the pandemic. Accordingly, this study aimed to evaluate the incidence of COVID-19 infection in asymptomatic children who were in close contact with symptomatic parents diagnosed with COVID-19 to provide evidence for reducing the household transmission rates of COVID-19.

## Methods

 The present cross-sectional study was conducted on 175 asymptomatic children who had been in close contact with confirmed COVID-19 cases. Confirmed cases in this study were identified through positive real-time RT-PCR tests on samples from upper respiratory nasopharyngeal swabs in Hamadan county from March 2021 to August 2021. The required sample size (175 patients) was determined based on an estimated asymptomatic COVID-19 infection rate of 35% among children according to the average of the estimated rate in the included studies to the review study by Gao et al, with a margin of error of 0.065 and a type 1 error equal to 0.05.^15^ The exclusion criteria included cases with no children aged 0-18 years in their family and children with a history of prior COVID-19 infection. RT-PCR was then performed on all asymptomatic children aged 0-18 years who were in close contact with a person with COVID-19, seven days after the family member tested positive for COVID-19.

 Close contacts were defined as individuals who were either family members or close relatives of the person with the disease who lived in the same house and had a close relationship with the index case up to two days before symptom onset.^16^

 The demographic characteristics of the patients, including gender, marital status, education level, age, residential location (urban or rural), and dwelling type (apartment or house, with specific details about the house’s size), were gathered through a researcher-developed questionnaire. Additionally, details regarding treatment type, underlying disease status, disease symptoms, and time from symptom onset to treatment initiation were meticulously recorded. Moreover, information about investigated children of family members with close contact with the index cases, including gender, age, education, hours of daily contact with the index case, and relationship type with the index case were gathered via semi-structured interviews with the child or guardian. Afterward, multiple logistic regressions with backward stepwise analysis were conducted to determine the predictors of COVID-19 transmission from family members to the children. Statistical analyses were also conducted using Stata version 16 (Stata Corp LP, College Station, TX, USA). Furthermore, a *P*-value less than 0.05 was considered statistically significant.

## Results

###  Characteristics of index cases

 In this study, 103 COVID-19 confirmed cases were investigated during the study period, of which 51.46% were male, and merely 11.65% possessed academic qualifications. Over 41% were aged 60 or older. Regarding accommodation, 66.02% resided in house courtyards. Furthermore, a significant number of cases (96.12%) exhibited symptoms, with almost 45% having underlying health conditions. Notably, 72.82% experienced a dry cough, and the average family size was 6.78n ± 4.44 members (Table 1).

**Table 1:** Characteristics of COVID-19 positive index cases (N = 103)

**Variables**	**Number**	**Percent**
Gender		
Male	53	51.46
Female	50	48.54
Age group (year)		
< 30	12	11.65
30-44.9	24	23.30
45.59.9	24	23.30
≥ 60	43	41.75
Education		
Illiterate	31	30.10
Less than diploma	44	42.72
Diploma	16	15.53
Academic	12	11.65
Type of accommodation		
Apartment	35	33.98
House courtyard	68	66.02
Disease symptom		
Symptomatic	99	96.12
Asymptomatic	4	3.88
Underlying disease		
No	58	56.31
Pulmonary disease	8	7.77
Cardiovascular disease	14	13.59
Diabetes	18	17.48
Cancer	3	2.91
Other	9	8.74
Dry cough		
Yes	75	72.82
No	28	27.18

###  Characteristics of children from family members with the coronavirus disease 2019 positive index cases

Table 2 presents the baseline characteristics of children from family members with COVID-19 positive index cases, of whom 50.86% were boys, and 41.71% were in the 6-12-year-old age group. Out of the 175 children in close contact with index cases, 53 (30.29%) tested positive for COVID-19 through PCR, while the PCR result was negative in 122 (69.71%) children. Moreover, family relationships with the index case were grandchild (44.57%) and child (44.57%). Furthermore, the means of daily contact hours with the index case was 11.09 ± 9.34 hours.

**Table 2:** Characteristics of investigated children from family members with COVID-19 positive index cases (N = 175)

**Variables**	**Number**	**Percent**
Gender		
Boy	89	50.86
Girl	86	49.14
Age group (year)		
0-5.9	53	30.29
6-11.9	73	41.41
12-18	49	28.00
Education		
Child	71	40.57
Less than diploma	88	50.29
Diploma	16	9.14
Family relationship with the index case		
Child	73	41.71
Grandchild	78	44.57
Brother/sister	13	7.43
Other	11	6.29
PCR result		
Positive	53	30.29
Negative	122	69.71

*Note.* PCR: Polymerase chain reaction.

###  Predictors of the COVID-19 transmission from family members to the children 

 Multivariable logistic regression analysis was used in Table 3 to identify predictors of COVID-19 transmission from family members to children. Notably, being the child, brother, or sister of the index case was found to be more strongly associated with disease transmission compared to being a grandchild (*P* < 0.05). Regarding factors related to the index case, male cases had higher odds of disease transmission to children compared to female cases (Adjusted odds ratio [AOR] = 2.29, 95% confidence interval [CI]: 1.03-5.09, *P* = 0.041). Additionally, the rural residency of the index case was significantly associated with increased odds of disease transmission to children (AOR = 3.22, 95% CI: 1.02-10.16, *P* = 0.046). Furthermore, cases presenting with nasal congestion symptoms were more prone to transmitting the virus to household contacts. Notably, the presence of this symptom in index cases was significantly associated with a positive PCR test result for COVID-19 in children who had close contact with them (AOR = 9.12, 95% CI: 2.22-37.40, *P* = 0.002). Moreover, index cases with limited literacy had higher odds of transmitting the virus to children (AOR = 8.45, 95% CI: 1.76-40.65, *P* = 0.008).

**Table 3:** Predictors of COVID-19 transmission from family members to the children

**Variables**	**Adjusted OR (95% CI)**	***P*** ** value**
**Factors related to the children in the family**		
Gender		
Girl	Ref.	
Boy	1.59 (0.79-3.02)	0.230
Family relationship with the index case		
Grandchild	Ref.	
Child	4.86 (1.02-23.25)	0.047
Brother/sister	4.62 (1.43-14.92)	0.011
Other	0.73 (0.67-8.00)	0.066
Age group (year)		
0-5.9	Ref.	
6-11.9	1.80 (0.71-4.61)	0.210
12-18	2.04 (0.77-5.40)	0.150
Hours of daily contact with the index case	1.04 (0.98-1.08)	0.170
**Factors related to the index case**		
Gender		
Female	Ref.	
Male	2.29 (1.03-5.09)	0.041
Education		
Diploma	Ref.	
Illiterate	8.45 (1.76-40.65)	0.008
Less than diploma	1.82 (0.57-5.75)	0.310
Academic	2.94 (0.71-12.10)	0.140
Location		
Urban	Ref.	
Rural	3.22 (1.02-10.16)	0.046
Runny nose		
No	Ref.	
Yes	9.12 (2.22-37.40)	0.002

*Note.* OR: Odds ratio; CI: Confidence interval.

## Discussion

 The results of the present study suggested that asymptomatic COVID-19 infection in household contacts was significant in children who were in close contact with a COVID-19-positive patient. Additionally, our analysis revealed that the transmission risk to children was significantly higher in male cases, individuals residing in rural areas, those with limited literacy, and those exhibiting nasal congestion symptoms.

 In a study conducted by Jing et al, the transmission rate to close contacts was estimated to be 19%.^17^ However, in the current study, the asymptomatic COVID-19 rate in close contact was 30.29%, indicating a higher risk of disease transmission in close contacts, which was not consistent with Jing and colleagues’^17^ study. Furthermore, the systematic review of the asymptomatic COVID-19 infections indicated that the rate of asymptomatic COVID-19 infection in different countries ranges from 1.6 to 56.5 which is in agreement with that of our study. It was further found that the rate of asymptomatic COVID-19 infection in children was 15.5, which is lower than that obtained in our study.^15^ Overall, all the studies published until 17 August 2021 investigating the role of asymptomatic COVID-19 infection and transmission reported that the asymptomatic COVID-19 transmission is 24.51%. Additionally, the abovementioned studies showed an asymptomatic COVID-19 transmission rate of 24.09% among children which is close to that in our study.^18^ The difference in incidence rate in different studies may be due to the distinct cultural context of the family in different countries and varying viral incubation periods among individuals. Furthermore, the presence of different serotypes of the virus across regions might play a role in shaping the epidemiological landscape as varying serotypes may exhibit distinct transmission patterns and severity levels. Recent surveillance data from the UK Office for National Statistics (ONS), based on random COVID-19 testing in households, revealed that relying on symptom-based testing significantly underestimates the actual incidence and prevalence of cases among children.^19^

 The current study revealed that male index patients are associated with an increased risk of transmitting COVID-19 to children. Interestingly, this finding contrasts with the results of Xin and colleagues’study,^20^ where female index patients were identified as having a higher risk of COVID-19 occurrence within households. The results of a meta-analysis on 229 studies involving over 10 million patients showed that males have a higher risk for COVID-19 infection and disease severity. Therefore, it seems that due to these conditions, males have a higher risk of disease transition in societies.^21^ Moreover, generally, women have higher adherence to COVID-19 isolation principles.^22^ However, such inconsistency highlights the complexity of factors affecting virus transmission dynamics and emphasizes the importance of considering varying demographic, geographic, and contextual factors in different research settings.

 Another finding of this study showed that parents with nasal congestion symptoms are more prone to COVID-19 transmission to close contacts. This finding is consistent with that of the study by Shah et al, suggesting that transmission might increase 3.2 times in this situation.^23^ The association between disease severity and the viral load of COVID-19 has been established in previous studies.^24^ Notably, individuals with serious or critical conditions tend to exhibit elevated viral loads in the respiratory tract compared to those with milder symptoms or asymptomatic cases and are more prone to disease transmission.

 Furthermore, it was found that individuals residing in rural areas with limited literacy have a higher risk of COVID-19 transmission to their children. This might be due to the lower level of self-care and awareness in this group with disease and less adherence to health protection measures which is consistent with the finding of Almalki and colleagues’ study which indicated a significant correlation between the degree of satisfaction with the COVID-19 preventive measures among public health students and the participant’s education level.^25^

 However, the present study had some limitations. Firstly, due to the presence of family members who might have had exposure outside the household, discerning whether their illness resulted from contact with the index case or exposure to other cases within the community poses a challenge. Secondly, establishing if a family member fell ill simultaneously with the index case but went undiagnosed as asymptomatic is complex. Finally, the presence of unidentified factors contributing to transmission, which act as potential confounders within the households of this novel disease, adds an element of uncertainty to our understanding.

**Highlights**

- Being the child, brother, or sister of the index case was more significantly associated with disease transmission.
- Male cases exhibited higher odds of the disease transmission to children compared to female cases.
- The rural residency of the index case was associated with an increased odds of disease transmission to children
- Cases presenting with nasal congestion symptoms were more prone to transmitting the virus to household contacts.
- Index cases with limited literacy exhibited higher odds of transmitting the virus to children.

## Conclusion

 Overall, the findings are consistent with those of previous studies that all children in contact with a family member infected with COVID-19 must observe for early isolation and social distancing. Furthermore, vaccination should be extensively performed in children in society to manage the missing gap of preventing COVID-19 transmission.

## Acknowledgements

 The authors would like to express their gratitude to the Hamadan University of Medical Sciences and the Ministry of Health, Iran, for financial support (Research ID: 990216769, Ethics code: IR.UMSHA.REC.1399.064). Additionally, the authors would like to acknowledge the efforts of Besat Hospital Research Development Unit and health staff in the Deputy of Health affiliated with the Hamadan University of Medical Sciences for their kind collaboration.

## Authors’ Contribution

**Conceptualization:** Iraj Sedighi, Jalaleddin Amiri.

**Data curation:** Roya Raeisi, Jalaleddin Amiri, Zohreh Shalchi, Farid Azizi Jalilian, Nastaran Ansari.

**Formal analysis:** Manoochehr Karami.

**Funding acquisition:** Iraj Sedighi.

**Investigation:** Iraj Sedighi, Roya Raeisi, Jalaleddin Amiri, Zohreh Shalchi, Ali Teimoori, Jalaledin Bathaei, Mohammad Hashemi.

**Methodology:** Manoochehr Karami.

**Project administration:** Iraj Sedighi, Roya Raeisi.

**Resources:** Iraj Sedighi, Jalaledin Bathaei.

**Software:** Manoochehr Karami.

**Supervision:** Iraj Sedighi.

**Visualization:** Iraj Sedighi.

**Writing–original draft:** Iraj Sedighi, Roya Raeisi, Jalaleddin Amiri, Zohreh Shalchi, Ali Teimoori.

**Writing–review & editing:** Iraj Sedighi, Jalaleddin Amiri, Farid Azizi Jalilian, Nastaran Ansari, Mohammad Hashemi.

## Competing Interests

 The authors declare that they have no competing interests.

## Ethical Approval

 The Ethics Committee of the Hamadan University of Medical Sciences approved the study protocol (Ethical Code: IR.UMSHA.REC.1399.064)

## Funding

 The Deputy of Research and Technology of Hamadan University of Medical Sciences funded this project (Research ID: 990216769).
